# Serum Angiopoietin-Like Protein 4: A Potential Prognostic Biomarker for Prediction of Vascular Invasion and Lymph Node Metastasis in Cholangiocarcinoma Patients

**DOI:** 10.3389/fpubh.2022.836985

**Published:** 2022-03-22

**Authors:** Tin May Aung, Mang Ngaih Ciin, Atit Silsirivanit, Apinya Jusakul, Worachart Lert-itthiporn, Tanakorn Proungvitaya, Sittiruk Roytrakul, Siriporn Proungvitaya

**Affiliations:** ^1^Faculty of Associated Medical Sciences, Centre of Research and Development of Medical Diagnostic Laboratories (CMDL), Khon Kaen University, Khon Kaen, Thailand; ^2^Cholangiocarcinoma Research Institute, Khon Kaen University, Khon Kaen, Thailand; ^3^Department of Biochemistry, Faculty of Medicine, Khon Kaen University, Khon Kaen, Thailand; ^4^Functional Ingredients and Food Innovation Research Group, National Center for Genetic Engineering and Biotechnology, National Science and Technology Development Agency, Pathum Thani, Thailand

**Keywords:** ANGPTL4, proteomics, bioinformatics, prediction, vascular invasion, lymph node metastasis, CCA

## Abstract

Cholangiocarcinoma (CCA) is a tumor arising from cholangiocytes lining the bile ducts. Vascular invasion and lymph node metastasis are important prognostic factors for disease staging as well as clinical therapeutic decisions for CCA patients. In the present study, we applied CCA sera proteomic analysis to identify a potential biomarker for prognosis of CCA patients. Then, using bioinformatics tools, we identified angiopoietin-like protein 4 (ANGPTL4) which expressed highest signal intensity among candidate proteins in proteomic analysis of CCA sera. Expression of ANGPTL4 in CCA tissues was determined using immunohistochemistry. The results showed that ANGPTL4 was stained at higher level in CCA cells when compared with normal cholangiocytes. The high expression of ANGPTL4 was associated with lymph node metastasis and advanced tumor stage (*p* = 0.013 and *p* = 0.031, respectively). Furthermore, serum ANGPTL4 levels in CCA and healthy control (HC) were analyzed using a dot blot assay. And it was found that ANGPTL4 level was significantly higher in CCA than HC group (*p* < 0.0001). ROC curve analysis revealed that serum ANGPTL4 level was effectively distinguished CCA from healthy patients (cutoff = 0.2697 arbitrary unit (AU), 80.0% sensitivity, 72.7% specificity, AUC = 0.825, *p* < 0.0001). Serum ANGPTL4 level was associated with vascular invasion and lymph node metastasis (*p* = 0.0004 and *p* = 0.006), so that it differentiated CCA with vascular invasion from CCA without vascular invasion (cutoff = 0.5526 AU, 64.9% sensitivity, 92.9% specificity, AUC = 0.751, *p* = 0.006) and it corresponded to CCA with/without lymph node metastasis (cutoff = 0.5399 AU, 71.4% sensitivity, 70.8% specificity, AUC = 0.691, *p* = 0.01) by ROC analysis. Serum ANGPTL4 levels showed superior predictive efficiency compared with CA 19-9 and CEA for vascular invasion and lymph node metastasis. In addition, serum ANGPTL4 level was an independent predictive indicator by multivariate regression analysis. In conclusion, serum ANGPTL4 could be a novel prognostic biomarker for prediction of vascular invasion and lymph node metastasis of CCA patients.

## Introduction

Cholangiocarcinoma (CCA) is a biliary malignancy arising from the bile duct cells (1). Its incidence is variable worldwide depending on the geographical risk factors as well as genetic variations (2). The high incidence of CCA is found in Southeast Asia especially in the Northeast Thailand (2) where the carcinogenic liver fluke infection (*Opisthorchis viverrini*), is a risk factor of CCA (3). The developmental stages of CCA based on tumor, node, metastasis (TNM) scoring system are: the tumor growths are found in intrahepatic bile duct without vascular invasion (stage I). The tumor growths are found in intrahepatic bile duct with vascular invasion or multiple tumors (stage II). The tumor has spread to visceral peritoneum (stage III). In the advanced tumor stage (stage IVA and IVB), the tumor has spread to lymph node or/and other parts of the body, such as bone, lungs, etc. (4). Surgery is the curative therapy for early stage of CCA patients, but most patients are diagnosed at advanced stage and chemotherapy is frequently used for those patients (5, 6). The median survival time for inoperable CCA patients is less than a year and the overall 5 years survival is <5% (7, 8). Such poor prognosis and high mortality rate of CCA patients, at least in part, due to late presentation of clinical symptoms (8). Vascular invasion and lymph node metastasis are important prognostic factors as well as reference factors in treatment of CCA patients (9, 10). By now, prognosis of CCA is confirmed by pathology and imaging techniques (9–13). However, these methods are invasive and required expertise hands with expensive tools. There are no serum markers currently recommended for diagnosis of vascular invasion and lymph node metastasis in CCA. Hence, it is critically necessary to identify a potential serum marker for poor prognosis CCA patients.

Recently, mass spectrometry-based serum proteomic analysis has been applied for biomarkers discovery for early detection of disease, measurement of disease progression, and discovery of new targets for treatment (14). In particular, proteins with secretory nature are likely to be potential biomarkers, and are important to differentiate secreted proteins from intracellular protein contaminants in serum/body fluid (15). For instance, apurinic/apyrimidinic endodeoxyribonuclease 1 (APEX-1) was a secreted protein which identified as a potential serum marker for diagnosis and prognosis of CCA (16). Moreover, other members of our group identified coiled-coil domain containing 25 (CCDC25), apoptosis-inducing factor, mitochondrion-associated 3 (AIFM3) and cytokine-induced apoptosis inhibitor 1 (CIAPIN1) as potential secreted protein biomarkers in CCA (17–19). In this study, we performed liquid chromatography-tandem mass spectrometry (LC-MS/MS) analysis of CCA patients' sera who were pathologically diagnosed with both vascular invasion and lymph node metastasis with ≤ 1 year survival time and >1 year survival time for searching a potential prognostic biomarker of CCA. Bioinformatics tools, Plasma Proteome Database (PPD), SignalP, and SecretomeP were used for the selection of candidate secreted proteins. Through those bioinformatic analysis, we have identified ANGPTL4 as a secreted protein, which expressed the highest signal intensity in CCA sera proteomic analysis. ANGPTL4 belongs to the angiopoietin (ANGPTL) family and the clinical and prognostic significance of serum ANGPTL4 has been reported in renal cell carcinoma (20). However, little research has been done on the circulation of ANGPTL4 in CCA patients. Therefore, in this study, ANGPTL4 expression was investigated in CCA patients and control group using immunohistochemistry and dot blot assay to find out whether it might be a potential prognostic biomarker of CCA patients.

## Materials and Methods

### Subjects and Sample Collection

CCA patients' sera pathologically diagnosed with vascular invasion and lymph node metastasis of ≤ 1 year survival time (Group A, *N* = 3) and >1 year survival time (Group B, *N* = 3) were used for LC-MS/MS analysis. The summary of CCA patients for LC-MS/MS is denoted in Supplementary Table 1. The surgically resected, formalin-fixed, paraffin-embedded tissues from intrahepatic CCA patients (*N* = 40) and preoperative sera of intrahepatic CCA (*N* = 90) with median age ± quartile deviation, range (61 ± 5, 31–83 years) were kindly given by Cholangiocarcinoma Research Institute (CARI), Faculty of Medicine, Khon Kaen University, Thailand. CCA patients who have been histopathologically diagnosed by intrahepatic CCA were included in this study whereas patients with extrahepatic or hepatocellular carcinoma were excluded. For normal controls, samples were collected from 44 healthy persons with median age ± quartile deviation, range (40 ± 7, 31–60 years) who had medical check-ups at AMS-KKU Excellence Laboratory, Faculty of Associated Medical Sciences, Khon Kaen University. Patients' sera involved in this study whose clinical demographic characteristics are presented in Supplementary Table 2. This study was approved by Human Ethics Committee of Khon Kaen University (HE631367) and informed consents were received from all participants.

### Sample Preparation and Trypsin Digestion

Three serum samples of Group A and Group B were individually diluted 1:20 with normal saline and protein concentration was measured by Bradford assay using bovine serum albumin as a standard. Five micrograms of each protein samples were subjected to in-solution digestion according to the method of the Functional Proteomics Technology Laboratory, National Center for Genetic Engineering and Biotechnology (BIOTEC), National Science and Technology Development Agency (NSTDA), Thailand. Briefly, samples were completely dissolved in 10 mM ammonium bicarbonate (AMBIC), then, 5 mM dithiothreitol (DTT) in 10 mM AMBIC was used for reducing disulfide bonds at 60°C for 1 hour (h) and 15 mM iodoacetamide (IAA) in 10 mM AMBIC was used for alkylation of sulfhydryl groups at room temperature (RT) for 45 min in the dark. Proteins were digested using sequencing grade trypsin (50 ng/μl) (1:20) (Promega, Germany) and incubated at 37°C overnight. Then, the digested samples were dried and resuspended in 0.1% formic acid before being injected into LC-MS/MS.

### LC-MS/MS Analysis

The digested samples were centrifuged at 10,000 × g for 10 min, and then injected into an Ultimate 3000 Nano/Capillary LC System (Thermo Scientific, UK) coupled to a Hybrid quadrupole Q-Tof impact II™ (Bruker Daltonics). Eluent A and B consisted of 0.1% formic acid and 80% acetonitrile in water containing 0.05% formic acid, respectively. Peptides were separated on a nanocolumn with a flow rate of 300 nl/min using elution period (3.0–30.0 min: 5–55% B), washing period (30.01–35.0 min: 95% B), and re-equilibration period (35.01–40.00 min: 1% B). The nanoLC system was connected to an electrospray ionization using the CaptiveSpray (Bruker Daltonik GmbH, Bremen, Germany). Mass spectra (MS) and MS/MS spectra were obtained in the positive-ion mode over the range of m/z 150–2,200 (Compass 1.9 for otofSeries software, Bruker Daltonics).

### Protein Quantitation and Identification

Proteins were quantified using Maxquant 1.6.6.0. (Maxquant, Independent Research Group Leader at the MPI of Biochemistry, Martinsried, Germany). The PepDetect module and Quantification were selected with peptide tolerance of 0.006 Da, intensity threshold of 30. The data files were sent against the NCBI human peptide database using the Andromeda software version 1.5.6 (Andromeda software Inc., California, US). Then, proteins were identified as at least three unique significant peptides that conserved in the known protein sequences in database.

### Selection of Candidate Proteins Using Bioinformatics Tools

Firstly, commons and unique proteins between Group A and Group B patients were investigated by Jvenn web tool (http://jvenn.toulouse.inra.fr/app/example.html, accessed on 14 January 2021) (21). Proteins were determined whether they have been reported in Plasma Proteome Database (PPD) (http://www.plasmaproteomedatabase.org, accessed on 14 January 2021) (22). Then, secretory protein nature was predicted using SignalP version 4.1 (Department of Bio and Health informatics, Technical University of Denmark, Lyngby, Denmark, accessed on 14 January 2021) and SecretomeP version 2.0 (Department of Bio and Health informatics, Technical University of Denmark, Lyngby, Denmark, accessed on 14 January 2021). SignalP was applied for prediction of signal peptides in the identified proteins (D-cutoff > 0.45 SignalP no transmembrane (TM) networks or > 0.5 SignalP TM networks as the default value for signal peptide = Yes) (23). SecretomeP 2.0 was used to predict a non-classical secretory protein which will be any proteins getting neural network (NN) score > 0.6 (24). Candidate secretory proteins were identified as proteins found in PPD with signal peptide and/or non-classical secretory proteins. Then, we selected the highest average mass spectrometry signal intensity of secreted protein for validation.

### Immunohistochemistry of ANGPTL4 Expression in Tissues

The 4 μm thickness section of paraffin-embedded tissues were deparaffinized and rehydrated using a series of graded xylene and alcohol. The slides were boiled in 0.01 M citrate buffer pH 6.0 for unmasking of antigenic epitopes in tissues. Then, endogenous peroxidase activity was blocked for 1 h at RT in the dark with 3% H_2_O_2_ in methanol and non-specific binding was blocked for 2 h at RT with 20% fetal bovine serum (FBS) in phosphate buffer saline with Tween 20 (PBS-T) (FBS, catalog No. 10270-098, Thermo Fisher scientific, USA). The slides were then incubated overnight with rabbit anti-ANGPTL4 polyclonal antibody (1:300; orb228886, Biorbyt, UK) at 4°C. Negative staining was incubated with the antibody diluent alone to determine the specificity of primary antibody. The slides were washed twice with 1X PBS-T, then they were incubated with secondary antibody (HRP labeled anti-Rabbit, EnVision+ system, Dako, Agilent Technologies, US) for 1 h. Peroxidase activity was detected using 3,3-diaminobenzidine tetrahydrochloride (DAB) substrate-chromogen system (1drop of liquid DAB chromogen in 1 ml of substrate buffer) (K-3468, Dako, Agilent Technologies, US) and counterstained with Mayer's hematoxylin. Finally, the slides were mounted with the coverslips using Permount™ (Thermo Fisher scientific, USA). The immunohistochemistry staining of ANGPTL4 was investigated using H-score method, in which both intensity level (0 = none; 1 = weak; 2 = moderate; and 3 = strong) and the percentage of stained cells were recorded and ranged according to H-score between 0 and 300 (25).

### Western Blot Analysis

To investigate the specificity of the ANGPTL4 antibody, 15 μg protein each randomly selected five of healthy controls and of CCA patients was separated on a 12.5% SDS-PAGE gel at 150 V for 2 h and electrically transferred to a polyvinylidenedifluoride (PVDF) membrane at 90 V for 2 h. The membrane was blocked for 1 h using 5% skim milk in Tris-buffered saline with 0.1% Tween-20 (1X TBST, pH 7.4) before being incubated overnight at 4°C with 1:2,000 ANGPTL4 (orb353642, Biorbyt, UK). After washing with 1X TBST, the membrane was incubated for 1 h at RT with 1: 10,000 goat anti-rabbit IgG secondary antibody conjugated with horseradish peroxidase. Then the membrane was washed with 1X TBST again. Finally, peroxidase activity was developed as chemiluminescence using an ECL solution (GE Healthcare Life Sciences, UK) and visualized under an Amersham imager 600. The pooled CCA serum was used as a loading control.

### Dot Blot Assay

Serum was diluted at 1:2 in normal saline and then 2 μl of diluted serum was spotted onto nitrocellulose membrane with Bio-Dot Microfiltration Apparatus (Bio-Rad, USA). All samples were spotted in random order on the membrane. The membrane was incubated with the same antibodies as that used for western blotting. The light intensity of each single spot on the membrane was developed as chemiluminescence using an ECL solution (GE Healthcare Life Sciences, UK) and visualized under an Amersham imager 600. Serum ANGPTL4 levels which indicated as the dot blot intensity of each spot was determined by ImageJ software v.1.52d (National Institute of Health, Bethesda, MD, USA). The positive control (pooled CCA serum) was put at the corner of the membrane in each assay. The serum ANGPTL4 levels were normalized using positive control (pooled CCA serum) and presented as arbitrary unit (AU). Samples were tested in triplicates.

### Enzyme-Linked Immunosorbent Assay

To correlate serum ANGPTL4 level between dot blot assay and ELISA, we determined ANGPTL4 level in randomly selected 20 samples using ANGPTL4 commercially quantitative sandwich ELISA kit (DY3485, R and D systems, USA) according to manufacturer's instructions. Serum was diluted 1:2 before detection. The ANGPTL4 concentration was calculated by reference to the standard curve. Samples were tested in triplicates.

### Statistical Analysis

The statistical analyses were conducted using IBM SPSS v.26 software (IBM Corp., Armonk, NY, USA). Data were showed as the median ± quartile deviation. The comparisons between CCA patient groups were analyzed using the Mann–Whitney *U*-test. Chi-square and Fisher's Exact test were used to determine the associations between ANGPTL4 and patients' clinicopathological parameters. The performance of ANGPTL4 was assessed by Receiver Operating Characteristic (ROC) curve analysis, Youden index (YI) value and subsequently the best cutoff levels of ANGPTL4 were selected to consider appropriate sensitivity and specificity. Parameters were analyzed using univariate and multivariate regression tests to determine relative risk for vascular invasion and lymph node metastasis. *p* < 0.05 was considered as statistical significance.

## Results

### CCA Sera Proteomics LC-MS/MS Analysis

**Figure 1:**
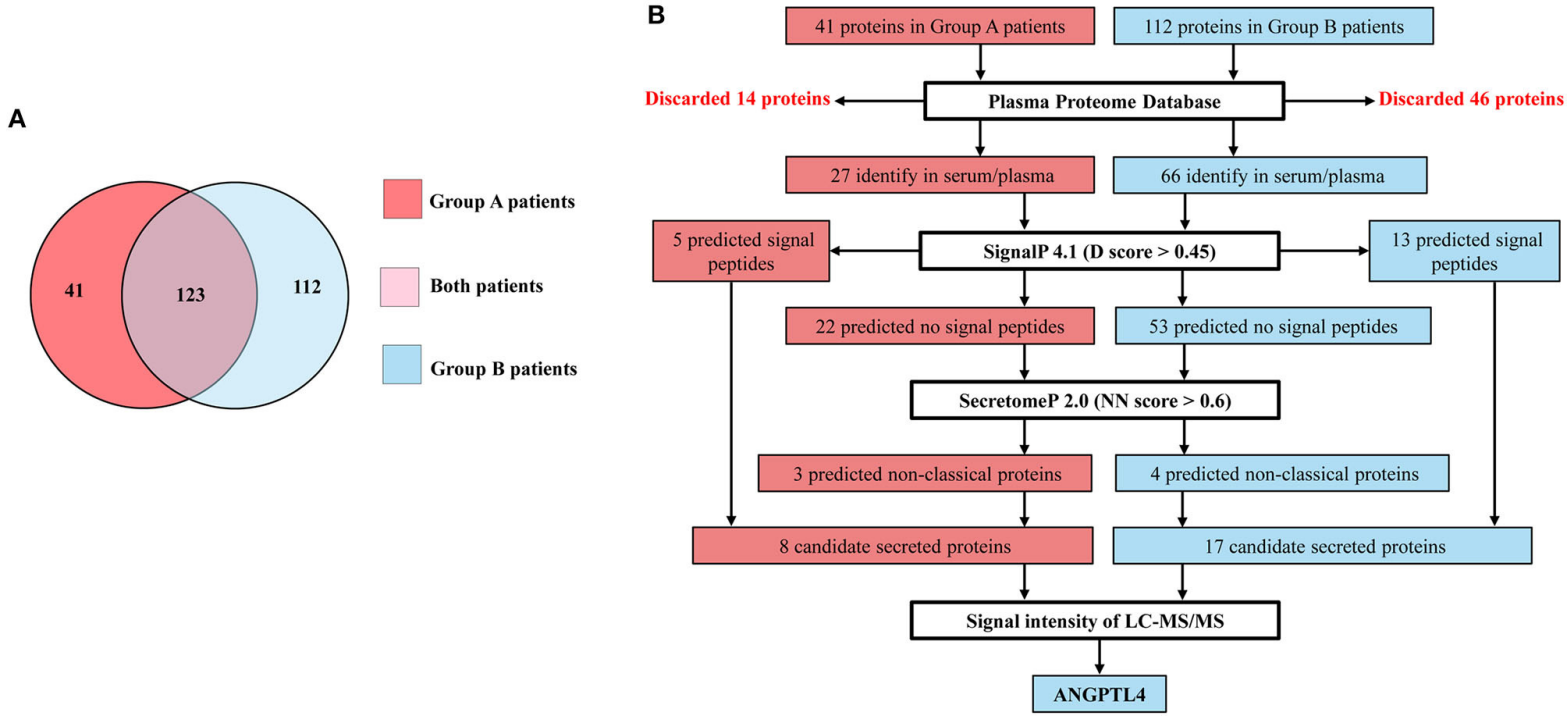
**(A)** Venn diagram showing 123 proteins express in both, 41 and 112 proteins uniquely express in Group A patients (CCA with ≤ 1 year survival time) and Group B patients (CCA with >1 year survival time). **(B)** Flow diagram of selection of candidate secreted proteins in Group A and Group B patients. Proteins identify in serum/plasma database and having signal peptide or non-classical proteins as candidate secreted proteins. ANGPTL4 is selected from Group B patients which express highest signal intensity of LC-MS/MS among candidate secreted proteins.

In order to identify the potential prognostics biomarker, we performed LC-MS/MS analysis of vascular invasion and lymph node metastasis CCA sera with ≤ 1 year survival time (Group A) and >1 year survival time (Group B). The LC-MS/MS analysis showed that a total of 5,442 proteins were identified with 95% CI, at least three unique significant peptides that conserved to the known protein sequences in database. Between 3,914 proteins were presented in Group A and 4,095 proteins were presented in group B patients. We further analyzed proteins which were expressed in all samples of each sera group with Q value cutoff ≥0.95 and *Homo sapiens*. Among them, 164 in Group A and 235 in Group B, were identified and then unique proteins of each group was examined using Jvenn software. According to Figure 1A, 41 and 112 proteins were uniquely expressed in Group A and Group B patients and the informations of proteins are summarized in Supplementary Tables 3, 4.

### Selection of Candidate Proteins Using Bioinformatics Tools

Since 41 and 112 proteins were uniquely expressed in Group A and Group B patients, candidate proteins were selected according to the flow diagram (Figure 1B). Firstly, 41 proteins from Group A and 112 proteins from Group B were screened in the Plasma Proteome Database (PPD) where the results showed that 27 were identified in Group A and 66 were identified in Group B. Then, further analyzing was done using SignalP, 4.1, to predict the presence of signal peptides and, 5 proteins in Group A and 13 proteins in Group B were predicted to be secreted proteins *via* classical secretory pathway. Then, whether the rest 22 in Group A and 53 proteins in Group B were examined in SecretomeP 2.0, 3 proteins in Group A and 4 proteins in Group B were predicted to be secretory proteins *via* non-classical secretory pathway. Finally, 8 proteins (5 secreted *via* classical secretory pathway + 3 secreted *via* non-classical secretory pathways) in Group A and 17 proteins (13 secreted *via* classical secretory pathway + 4 secreted *via* non-classical secretory pathways) in Group B were identified as candidate secreted proteins. Among them, ANGPTL4 was selected for validation from Group B patients because it has the highest signal intensity of LC-MS/MS analysis (Supplementary Figure 1).

### ANGPTL4 Expression in CCA Tissues

**Figure 2:**
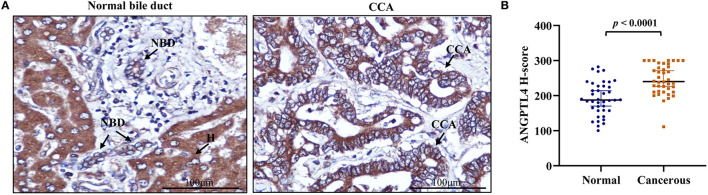
**(A)** Representative immunohistochemical staining of ANGPTL4 in CCA tissues (magnification, x400). Hepatocytes (H) strongly express ANGPTL4 while normal bile duct (NBD) express weak positive staining and CCA cells express strongly positive staining. **(B)** H-scores of ANGPTL4 in normal and CCA tissues using immunohistochemistry. Mann-Whitney *U*-test was used to compare H-scores between normal and cancerous tissues of ANGPTL4. Statistical significance (*p* < 0.05).

To investigate ANGPTL4 expression in CCA tissues, we performed immunohistochemistry of surgically resected, paraffin-embedded tissues from intrahepatic CCA patients (*N* = 40). The results revealed that ANGPTL4 was strongly stained in hepatocytes and with lesser intensity in normal intrahepatic bile duct epithelia. The staining intensity of ANGPTL4 was higher in tumor cells when compared with normal bile duct epithelia (Figure 2A). The median H-score of ANGPTL4 in cancerous tissues (240 ± 39.12) was significantly higher than that found in those of normal tissues (187 ± 69.75) (Figure 2B). To examine the clinical importance of ANGPTL4, the median H-score value 240 of 40 cancerous tissues was used as the cutoff to discriminate low and high ANGPTL4 expression group and analyzed by Chi-square and Fisher's Exact test. As shown in Table 1, high ANGPTL4 expression was significantly correlated with lymph node metastasis and advanced tumor stage (*p* = 0.013 and *p* = 0.031, respectively).

**Table 1:** Association between ANGPTL4 expression and clinicopathological data of CCA patients.

**Category**	**Number of patients**	**ANGPTL4 expression H-score**		***P*-value**
		**Low expression ≤240**	**High expression > 240**	
**Gender**				
Male	28	14 (35%)	14 (35%)	1
Female	12	6 (15%)	6 (15%)	
**Age (years)**				
≤ 61	21	9 (22.5%)	12 (30%)	0.399
>61	19	11 (27.5%)	8 (20%)	
**Vascular invasion**				
No	4	1 (4.2%)	3 (12.5%)	0.36
Yes	20	10 (41.7%)	10 (41.7%)	
**Lymph node metastasis**				
No	9	7 (23.3%)	2 (6.7%)	0.013^*^
Yes	21	5 (16.7%)	16 (53.3%)	
**Tumor stage**				
I–III	10	8 (20.5%)	2 (5.1%)	0.031^*^
IVA–IVB	29	11 (28.2%)	18 (46.2%)	
**Histopathological grading**				
Papillary	16	6 (15%)	10 (25%)	0.197
Non-papillary	24	14 (35%)	10 (25%)	
**Survival (days)**				
≤ 563	20	10 (25%)	10 (25%)	1
>563	20	10 (25%)	10 (25%)	

*Parameters and ANGPTL4 expression of tissues were analyzed using Chi-square and Fisher's Exact test. Total 40 patients were not fully determined due to absent of the corresponding clinical data. Bold values*

* *Statistical significance (p <0.05)*.

### ANGPTL4 Levels in the Sera of Healthy Controls and CCA Patients

**Figure 3:**
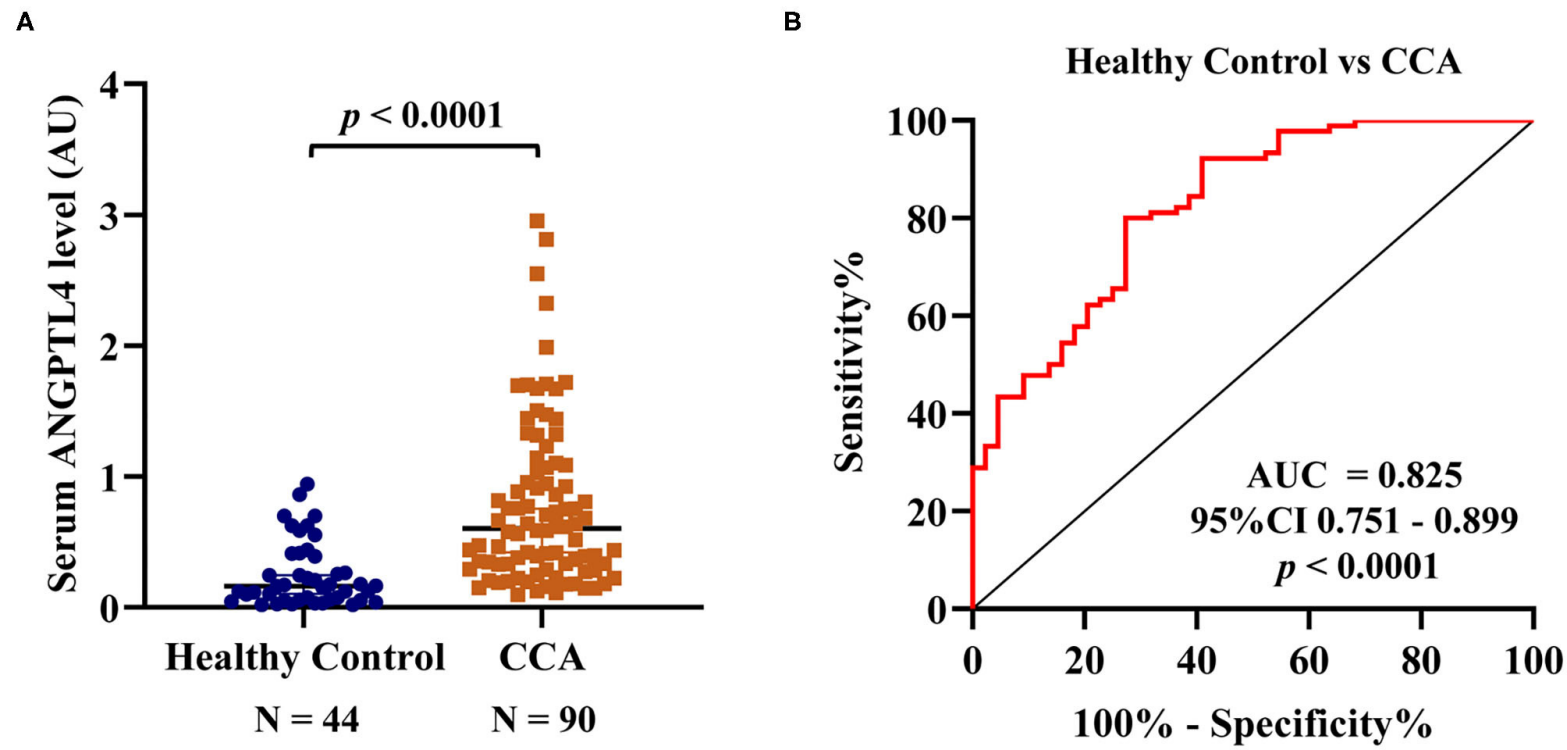
**(A)** Serum ANGPTL4 level of HC and CCA patients by dot blot assay. The median ± quartile deviation, 0.1634 ± 0.1739 AU (0.0193–0.9424 AU), in healthy control and 0.6017 ± 0.3767 AU (0.0984–2.9526 AU) in CCA patients' group. To normalize the expression level, the ratio of the intensity of each dot blot to that of the positive control (pooled CCA serum) was calculated and determined as the arbitrary unit (AU). The difference of serum ANGPTL4 level between HC and CCA by Mann-Whitney U-test. **(B)** ROC curve of serum ANGPTL4 level as a potential biomarker for prediction of CCA in comparison group of HC, sensitivity = 80.0% and specificity = 72.7% (AUC = 0.825, 95% CI 0.751–0.899, *p* < 0.0001). Statistical significance (*p* < 0.05).

Firstly, to check the specificity of the anti-ANGPTL4 antibody, ANGPTL4 protein expression was investigated in randomly selected five sera of CCA and healthy patients using Western blot analysis. Clear single bands of ~50 kDa, corresponding to the molecular weight of ANGPTL4 was found in CCA and HC sera including pooled CCA serum control of dot blot. Also, the serum ANGPTL4 levels were high in CCA (Supplementary Figure 2). Then, the serum ANGPTL4 level was examined using larger numbers of CCA (*N* = 90) and HC (*N* = 44) using a dot blot assay. Pooled CCA serum was used as normalization (positive) control in every determination (Supplementary Figure 3). The results showed that serum ANGPTL4 level in CCA patients (0.6017 ± 0.3767 AU) was significantly higher than that of healthy controls (0.1634 ± 0.1739 AU, *p* < 0.0001) (Figure 3A). The predictive value of ANGPTL4 was analyzed by ROC curve and area under curve (AUC). According to ROC curve analysis, ANGPTL4 could be applied as a biomarker to differentiate between HC and CCA patients with cutoff ≤ 0.2697 AU, sensitivity = 80.0% and specificity = 72.7% (AUC = 0.825, 95% CI 0.751–0.899, YI = 0.527, *p* < 0.0001) (Figure 3B). Moreover, the correlation of ANGPTL4 level between dot blot assay and ELISA (N =20) was examined. The results showed that ANGPTL4 level (AU) obtained by dot blot assay and ANGPTL4 concentration (ng/mL) obtained by ELISA were positively correlated (r = 0.9203, *p* < 0.0001) (Supplementary Figure 4).

### Association Between Serum ANGPTL4 Levels and Clinicopathological Parameters of CCA Patients

To investigate the clinical importance of ANGPTL4, the correlation between serum ANGPTL4 and clinicopathological features of CCA patients was investigated using dot blot assay. CCA patients were dichotomized into low and high level of ANGPTL4 using the median cutoff value 0.6017 AU. Then, the association between serum ANGPTL4 level and clinicopathological parameters was analyzed by Chi-square and Fisher's Exact test. The results showed that high serum ANGPTL4 level was significantly associated with vascular invasion (*p* = 0.0004) and lymph node metastasis (*p* = 0.006). However, there was no significant association of serum ANGPTL4 level and gender, age, tumor stage, and survival days (Table 2).

**Table 2:** Association between serum ANGPTL4 level and clinicopathological parameters in CCA patients.

**Category**	**Number of patients**	**Serum ANGPTL4 level (AU)**		***P*-value**
		**Low level ≤0.6017**	**High level >0.6017**	
**Gender**				
Male	60	30 (33.33%)	30 (33.33%)	1
Female	30	15 (16.66%)	15 (16.66%)	
**Age (years)**				
≤ 61	46	21 (23.3%)	25 (27.8%)	0.399
>61	44	24 (26.7%)	20 (22.2%)	
**Vascular invasion**				
No	14	13 (25.5%)	1 (2%)	0.0004^*^
Yes	37	14 (27.5%)	23 (45.1%)	
**Lymph node metastasis**				
No	24	17 (25.8%)	7 (10.6%)	0.006^*^
Yes	42	15 (22.7%)	27 (40.9%)	
**Tumor stage**				
I–III	33	21 (24.4%)	12 (14%)	0.097
IVA–IVB	53	24 (27.9%)	29 (33.7%)	
**Histopathological grading**				
Papillary	38	23 (27.1%)	15 (17.6%)	0.208
Non-papillary	47	22 (25.9%)	25 (29.4%)	
**Survival (days)**				
≤ 708	45	20 (22.5%)	25 (28.1%)	0.341
>708	44	24 (27%)	20 (22.5%)	
**ALT (U/L)**				
≤ 37	42	23 (27.7%)	19 (22.9%)	0.586
>37	41	20 (24.1%)	21 (25.3%)	
**AST (U/L)**				
≤ 42	45	24 (28.6%)	21 (25%)	0.673
>42	39	19 (22.6%)	20 (23.8%)	
**ALP (U/L)**				
≤ 168.5	42	20 (23.8%)	22 (26.2%)	0.513
>168.5	42	23 (27.4%)	19 (22.6%)	
**CA 19-9 (U/mL)**				
≤ 120.8	39	22 (28.6%)	17 (22.1%)	0.306
>120.8	38	17 (22.1%)	21 (27.3%)	
**CEA (ng/mL)**				
≤ 5.44	35	20 (28.6%)	15 (21.4%)	0.473
>5.44	35	17 (24.3%)	18 (25.7%)	

*Parameters and serum ANGPTL4 level were analyzed by Chi-square test. Total 90 patients were not fully determined due to absent of the corresponding clinical data*.

* *Statistical significance (p <0.05)*.

### Predictive Value of Serum ANGPTL4 in Comparisons With CA 19-9 and CEA Levels for Vascular Invasion and Lymph Node Metastasis

**Figure 4:**
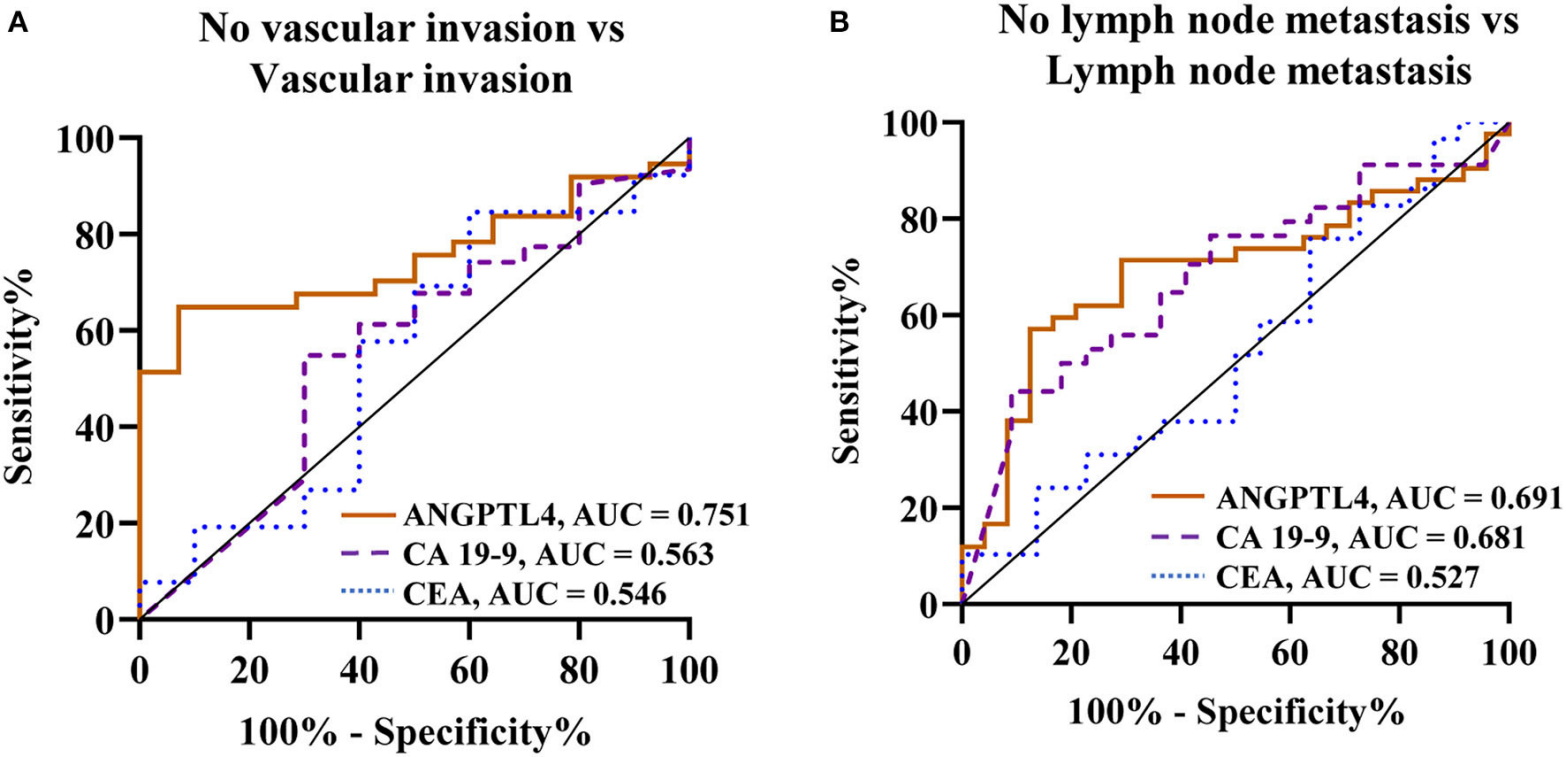
ROC curve analysis of biomarkers (ANGPTL4, CA 19-9, and CEA) for prediction of CCA in comparison group of **(A)** No vascular invasion vs. Vascular invasion, **(B)** No lymph node metastasis vs. Lymph node metastasis. Bold values ^*^Statistical significance (*p* < 0.05).

To determine the predictive values of vascular invasion and lymph node metastasis in CCA patients, we analyzed ANGPTL4 levels and current tumor markers, CA 19-9 and CEA between patient groups by Mann-Whitney U-test and ROC analysis (Figures 4A,B, Table 3, Supplementary Figures 5A–F). The results showed that ANGPTL4 level was only significant higher in CCA patients with vascular invasion than in those without vascular invasion (*p* = 0.004). ANGPTL4 level could be used to differentiate patients with vascular invasion from those without vascular invasion, with the cutoff 0.5526 AU, 64.9% sensitivity and 92.9% specificity (AUC = 0.751, YI = 0.577, *p* = 0.006). Moreover, serum ANGPTL4 and CA 19-9 levels were significantly higher in CCA patients with lymph node metastasis than in those without lymph node metastasis (*p* = 0.008 and *p* = 0.023). The ROC analysis showed that ANGPTL4 could be used to predict lymph node metastasis patients with the cutoff 0.5399 AU, sensitivity of 71.4% and specificity of 70.8% (AUC = 0.691, YI = 0.423, *p* = 0.01). Furthermore, CA 19-9 levels could be used to predict lymph node metastasis patients with the cutoff 212.6 U/mL, sensitivity of 52.9% and specificity of 77.3% (AUC = 0.681, YI = 0.302, *p* = 0.023), respectively. By contrast, CEA does not have prognostic value for predicting the vascular invasion and lymph node metastasis in CCA patients.

**Table 3:** Predictive values of serum ANGPTL4, CA 19-9, and CEA levels for vascular invasion and lymph node metastasis of CCA patients, based on the optimal cutoff derived from ROC analysis and YI calculation.

**Tumor markers**	**Cutoff**	**AUC**	**Sensitivity (%)**	**Specificity (%)**	**YI**	***P*-value**
**No vascular invasion vs. vascular invasion**						
ANGPTL4 (AU)	0.5526	0.751	64.9	92.9	0.577	0.006^*^
CA 19-9 (U/mL)	162.2	0.563	54.8	70.0	0.248	0.554
CEA (ng/mL)	3.18	0.546	69.2	50.0	0.192	0.672
**No lymph node metastasis vs. lymph node metastasis**						
ANGPTL4 (AU)	0.5399	0.691	71.4	70.8	0.423	0.01^*^
CA 19-9 (U/mL)	212.6	0.681	52.9	77.3	0.302	0.023^*^
CEA (ng/mL)	2.92	0.527	75.9	36.4	0.122	0.746

*AUC, Area under the ROC curve; YI, Youden index; Bold values*

* *Statistical significance (p <0.05)*.

### Univariate and Multivariate Regression Analysis of Clinical Parameters Associated With Vascular Invasion and Lymph Node Metastasis

To identify independent predictors of vascular invasion and lymph node metastasis of CCA patients, we conducted univariate regression analysis of the following covariates: gender, age, ALT, AST, ALP, CA 19-9, CEA, and ANGPTL4. We found that vascular invasion was associated with elevated ALT (RR = 4.524, *p* = 0.043) and ANGPTL4 level (RR = 21.357, *p* = 0.005). However, multivariate analysis showed that ANGPTL4 was an independent marker for predicting vascular invasion of CCA patients (RR = 72.182, *p* = 0.025) (Table 4). Moreover, ANGPTL4 was identified as an independent marker for predicting lymph node metastasis in both univariate and multivariate analysis (RR = 4.371, *p* = 0.008 and RR = 3.65, *p* = 0.039) (Table 5).

**Table 4:** Univariate and multivariate regression analysis of clinical parameters associated with vascular invasion.

**Variables**	**Univariate analysis**			**Multivariate analysis**		
	**RR**	**95% CI**	***P*-value**	**RR**	**95% CI**	***P*-value**
**Gender**						
Male	1			1		
Female	2.231	0.529–9.412	0.666	3.342	0.309–36.184	0.321
**Age (years)**						
≤ 61	1			1		
>61	0.762	0.222–2.615	0.274	9.164	0.464–181.157	0.146
**ALT (U/L)**						
≤ 37	1			1		
>37	4.524	1.047–19.543	0.043^*^	20.321	0.851–485.089	0.063
**AST (U/L)**						
≤ 42	1			1		
>42	1.263	0.342–4.665	0.726	0.272	0.015–4.8	0.374
**ALP (U/L)**						
≤ 168.5	1			1		
>168.5	0.921	0.255–3.324	0.9	0.968	0.099–9.509	0.978
**CA 19-9 (U/mL)**						
≤ 120.8	1			1		
>120.8	2.077	0.486–8.876	0.324	0.705	0.078–6.411	0.705
**CEA (ng/mL)**						
≤ 5.44	1			1		
>5.44	1.75	0.398–7.7	0.459	8.317	0.515–134.201	0.135
**ANGPTL4 (AU)**						
≤ 0.6017	1			1		
>0.6017	21.357	2.513–181.471	0.005^*^	72.182	1.732–3007.726	0.025^*^

*RR, relative risk; CI, confidence interval; Bold values*

* *Statistical significance (p <0.05)*.

**Table 5:** Univariate and multivariate regression analysis of clinical parameters associated with lymph node metastasis.

**Variables**	**Univariate analysis**			**Multivariate analysis**		
	**RR**	**95% CI**	***P*-value**	**RR**	**95% CI**	***P*-value**
**Gender**						
Male	1			1		
Female	0.897	0.307–2.618	0.842	1.25	0.35–4.461	0.731
**Age (years)**						
≤ 61	1			1		
>61	0.886	0.323–2.431	0.815	1.287	0.357–4.638	1.287
**ALT (U/L)**						
≤ 37	1					
>37	1.606	0.566–4.559	0.374	–	–	–
**AST (U/L)**						
≤ 42	1					
>42	1.606	0.566–4.559	0.374	–	–	–
**ALP (U/L)**						
≤ 168.5	1					
>168.5	1.132	0.401–3.198	0.815	–	–	–
**CA 19-9 (U/mL)**						
≤ 120.8	1			1		
>120.8	2.827	0.931–8.581	0.067	2.156	0.612–7.596	0.232
**CEA (ng/mL)**						
≤ 5.44	1			1		
>5.44	0.893	0.294–2.712	0.842	0.986	0.268–3.63	0.983
**ANGPTL4 (AU)**						
≤ 0.6017	1			1		
>0.6017	4.371	1.480–12.913	0.008^*^	3.65	1.068–12.472	0.039^*^

*RR, relative risk; CI, confidence interval; Bold values*

* *Statistical significance (p <0.05)*.

## Discussion

The prognosis of CCA is depending on vascular invasion, lymph node metastasis and tumor grade even after curative resection (26), however, there have been few studies on prognostics parameters in CCA patients (9, 10). Therefore, identification of biomarker associated with tumor progression which is helpful for preoperative risk assessment, metastasis monitoring and guiding treatment decisions. In the present study, we used LC-MS/MS technique on CCA sera to identify a potential prognostics biomarker of CCA. Using bioinformatic analysis of PPD, SignalP, and SecretomeP, we have compared protein expression patterns of the sera from CCA patients with >1 year survival time and those in the sera of CCA patients with ≤ 1 year survival time with vascular invasion and lymph node metastasis. Among candidate proteins expressed higher in the sera of longer survival than in the sera of shorter survival, we have selected ANGPTL4 having the highest signal intensity for validation. Hence, ANGPTL4 is suggested to be a potential serum marker for some cancers (20, 27), but its expression in tissues and serum of CCA patients has not yet been studied. Therefore, in this study, we investigated ANGPTL4 expression levels in control and CCA patients using immunohistochemistry and dot blot assay.

ANGPTL4, a member of ANGPTL family (ANGPTL1-8), has a full length of 45–65 kDa and is abundant in adipose tissues, vascular system, liver, and intestine, etc. (28). Interestingly, ANGPTL4 has been involved in glucose and lipid metabolisms, inflammation, differentiation, angiogenesis, and tumorigenesis (29, 30). In the current study, immunohistochemistry results showed that ANGPTL4 expression was higher in CCA tissues than in normal cholangiocytes (*p* < 0.0001). The high expression of ANGPTL4 in cancer tissues was significantly associated with lymph node metastasis and advanced tumor stage (*p* = 0.013 and *p* = 0.031). This finding was consistent with other studies in which ANGPTL4 expression was associated to metastasis-related clinicopathological characteristics in tongue squamous cell carcinoma, colorectal cancer, cervical cancer and breast cancer (31–34) indicating that ANGPTL4 might play a role in the metastasis of human cancer. In the present study, serum ANGPTL4 level was significantly elevated in CCA patients compared with healthy controls (*p* < 0.0001). Similar to our results, previous studies (27, 35) revealed higher level of ANGPTL4 in the sera of patients with hepatocellular carcinoma and esophageal squamous cell carcinoma. Our dot blot assay showed that high level of serum ANGPTL4 was associated with vascular invasion and lymph node metastasis (*p* = 0.0004 and *p* = 0.006), but not associated with survival time. The discrepancy between the results of LC-MS/MS and dot blot findings of the survival time could be primarily attributed to the difference in the numbers of serum samples. In proteomic study, only 3 representative serum samples of CCA patients with longer and shorter survival were used, whereas in the dot blot assay, 90 serum samples were examined together with 44 healthy control sera. As shown in Figure 3A, apparently more than half of CCA patients have serum ANGPTL4 level comparable with healthy controls regardless of their survival time. Related to this, in this study, ANGPTL4 expression in CCA tissues correlated with lymph node metastasis and tumor stage but did not correlate with vascular invasion. Serum ANGPTL4 showed a correlation with vascular invasion and lymph node metastasis but did not correlate with tumor stage. Such a discrepancy might be at least due in part to the difference of the samples. Immunohistochemical detection of ANGPTL4 in CCA cells represented production of this protein in CCA cells only, where as serum level might reflect not only the protein produced by CCA cells but also by normal cells such as hepatocytes. Also, such discrepancy might be due to the fact that the function of ANGPTL4 in cancer progression may be determined by CCA subtypes. Moreover, the primary source and the tumor microenvironment of ANGPTL4 may impact the biological functions.

In this study, serum ANGPTL4 levels in CCA patients were found to be a better predictor of vascular invasion (cutoff = 0.5526 AU, sensitivity = 64.9%, specificity = 92.9%, AUC = 0.751) and lymph node metastasis (cutoff = 0.5399 AU, sensitivity = 71.4%, specificity = 70.8%, AUC = 0.691) than CA 19-9 and CEA. Furthermore, we studied univariate and multivariate of regression analyses to investigate clinical parameter variables which could be potentially related to vascular invasion and lymph node metastasis. Univariate analysis showed that elevated ALT and ANGPTL4 level were associated to vascular invasion in CCA patients. Multivariate analysis revealed that serum ANGPTL4 level was an independent predictor of vascular invasion. Additionally, serum ANGPTL4 levels were identified as an independent predictor of lymph node metastasis in univariate and multivariate analyses.

Previous studies reported that ANGPTL4 disrupt the endothelial cell-cell junction and enhance the tumor cells infiltration into adjacent capillary which may begin distant metastasis to other organs through blood flow (36). ANGPTL4 promotes tumor metastasis by signaling through the transforming growth factor β (TGF-β) pathway (37). Moreover, emerging evidence indicates that ANGPTL4 expression, induced by hypoxia-inducible factor 1 alpha (HIF1α) under hypoxic conditions, promotes tumor cells aggressiveness and metastasis (38, 39). One report revealed an additional tumor-promoting activity of ANGPTL4 by interacting with integrin 1/5, which increases O2- levels, and activates the Src, PI3K/PKB, and ERK pathways. The activation of this signaling pathway confers anoikis resistance, which promotes tumor growth (40). Recent study of San et al. found that ANGPTL4 was upregulated in AI-CCA cells (anchorage-independently cultured), and that its absence caused CCA cells death (41). Thus, our study confirmed that ANGPTL4 might be involved in tumor progression and metastasis of CCA, resulting in poor prognosis. However, other investigations will be needed to define the functions of ANGPTL4 in CCA.

## Conclusions

Our data showed that high expression of ANGPTL4 in CCA tissues was significantly correlated with advanced tumor stage and metastasis. Moreover, higher level of ANGPTL4 in CCA sera could be used to differentiate CCA with vascular invasion and lymph node metastasis from without these complications. Multivariate analysis showed that serum ANGPTL4 level could be an independent serum marker for prognostics prediction of CCA patients. Therefore, serum ANGPTL4 might be a novel prognostic marker with a significant impact on risk of vascular invasion and lymph node metastasis of CCA patients.

## Data Availability Statement

The original contributions presented in the study are included in the article/Supplementary Material, further inquiries can be directed to the corresponding author/s.

## Ethics Statement

The studies involving human participants were reviewed and approved by Human Ethics Committee of Khon Kaen University (HE631367). The patients/participants provided their written informed consent to participate in this study.

## Author Contributions

AS, TP, WL-i, AJ, and SP: conceptualization. MC and TA: methodology. SR and TA: software. TA and SP: investigation. TA and AS: data curation. TA: writing—original draft. AS, TP, AJ, and SP: writing—review and editing. SP: funding acquisition. All authors have read and agreed to the published version of the manuscript, contributed to the article, and approved the submitted version.

## Funding

This research was supported by Centre of Research and Development of Medical Diagnostic Laboratories (CMDL), Faculty of Associated Medical Sciences, Khon Kaen University, and the Research Grant, Faculty of Associated Medical Sciences, Khon Kaen University.

## Conflict of Interest

The authors declare that the research was conducted in the absence of any commercial or financial relationships that could be construed as a potential conflict of interest. The reviewers TB and SK declared a shared affiliation, with no collaboration, with several of the authors TA, MC, AS, AJ, WL-i, TP, and SP to the handling editor at the time of the review.

## Publisher's Note

All claims expressed in this article are solely those of the authors and do not necessarily represent those of their affiliated organizations, or those of the publisher, the editors and the reviewers. Any product that may be evaluated in this article, or claim that may be made by its manufacturer, is not guaranteed or endorsed by the publisher.
