# Supplementary material for: Serum Angiopoietin-Like Protein 4: A Potential Prognostic Biomarker for Prediction of Vascular Invasion and Lymph Node Metastasis in Cholangiocarcinoma Patients
Source: Front Public Health. 2022 Mar 22;10:836985. doi: 10.3389/fpubh.2022.836985 (PMC8980351; doi:10.3389/fpubh.2022.836985)
Supplement: Supplementary file 1 [file Table_1.DOCX]

Supplementary Material

**Table S1.** Summary of CCA Patients Collected for LC-MS/MS

|  | **Group A (N=3)** | **Group B (N=3)** |
| --- | --- | --- |
| Age (years) | 61±4 (58-66) | 57±2 (54-58) |
| Gender (Male:Female) | 3:0 | 2:1 |
| ALT (U/L) | 22±5.57 (16-27) | 48±16.04 (33-65) |
| AST (U/L) | 26±5.13(22-32) | 38±6.43 (33-45) |
| ALP (U/L) | 145±66.01 (72-200) | 201±27 (174-228) |
| Vascular invasion | Yes | Yes |
| Lymph node metastasis status | Yes | Yes |
| Papillary:Non-papillary | 2:1 | 0:3 |
| Survival days | 108 ± 73 (46-188) | 823 ± 400 (485-1264) |

Values are given as mean ± standard deviation (min-max)
