# Supplementary material for: Serum Angiopoietin-Like Protein 4: A Potential Prognostic Biomarker for Prediction of Vascular Invasion and Lymph Node Metastasis in Cholangiocarcinoma Patients
Source: Front Public Health. 2022 Mar 22;10:836985. doi: 10.3389/fpubh.2022.836985 (PMC8980351; doi:10.3389/fpubh.2022.836985)
Supplement: Supplementary file 2 [file Table_2.DOCX]

Supplementary Material

**Table S2.** Clinical demographic characteristics of HC and CCA patients

|  | **HC (N = 44)** | **CCA (N = 90)** | **P value** |
| --- | --- | --- | --- |
| Gender (Male:Female) | 10:34 | 60:30 | NA |
| Age (years) | 40±7 (31-60) | 61±5 (31-83) | <0.001* |
| ALT (U/L) | 16±5 (7-42) ^a^ | 37±21 (9-257) ^d^ | <0.001* |
| AST (U/L) | 21±3.5 (16-30) ^a^ | 42±18.25 (14-280) ^e^ | <0.001* |
| ALP (U/L) | 50±8.38 (32-86) ^b^ | 168.5±87.5 (35-712) ^e^ | <0.001* |
| CA 19-9 (U/mL) | NA | 120.8±421.61 (0.6-1000) ^f^ | NA |
| CEA (ng/mL) | 1.57± 1.15 (0.67-6.93) ^c^ | 5.44±5.95 (1-917.6) ^g^ | <0.001* |

Values are given as median ± quartile deviation (min–max), a, b, c represented the number of analyzed samples in HC (healthy control) =43, 42, 17 and d, e, f, and g represented the number of analyzed samples in CCA (cholangiocarcinoma) = 83, 84, 77 and 70 respectively, NA = not analyzed, ALT = alanine transaminase, AST = aspartate transaminase, ALP = alkaline phosphatase, CA 19-9 = carbohydrate antigen 19-9, CEA = carcinoembryonic antigen. The difference between groups were calculated by Mann-Whitney U test. * Statistical significance (*p* < 0.05).
