# Supplementary material for: Serum Angiopoietin-Like Protein 4: A Potential Prognostic Biomarker for Prediction of Vascular Invasion and Lymph Node Metastasis in Cholangiocarcinoma Patients
Source: Front Public Health. 2022 Mar 22;10:836985. doi: 10.3389/fpubh.2022.836985 (PMC8980351; doi:10.3389/fpubh.2022.836985)
Supplement: Supplementary file 3 [file Table_3.DOCX]

Supplementary Material

**Table S3.** Identified proteins in Group A patients of LC-MS/MS analysis

| **Uniport accession number** | **Gene symbol** | **Protein name** | **Average signal intensity** |
| --- | --- | --- | --- |
| P42263 | GRIA3 | Glutamate receptor 3 | 80278.2 |
| Q92576 | PHF3 | PHD finger protein 3 | 33093.4 |
| Q9H8Y5 | ANKZF1 | Ankyrin repeat and zinc finger domain-containing protein 1 | 76637.33 |
| Q8IYS4 | DNAAF8 | Dynein axonemal assembly factor 8 | 55267.1 |
| O14792 | HS3ST1 | Heparan sulfate glucosamine 3-O-sulfotransferase 1 | 115249.5 |
| Q9BVQ7 | SPATA5L1 | Spermatogenesis-associated protein 5-like protein 1 | 178441.3 |
| P49137 | MAPKAPK2 | MAP kinase-activated protein kinase 2 | 49867.67 |
| Q8NDM7 | CFAP43 | Cilia- and flagella-associated protein 43 | 300975.3 |
| Q96JH8 | RADIL | Ras-associating and dilute domain-containing protein | 93909 |
| O75820 | ZNF189 | Zinc finger protein 189 | 350144.3 |
| Q8ND61 | C3orf20 | Uncharacterized protein C3orf20 | 32726.33 |
| Q9UHI6 | DDX20 | Probable ATP-dependent RNA helicase DDX20 | 517632.7 |
| O94827 | PLEKHG5 | Pleckstrin homology domain-containing family G member 5 | 39507.67 |
| Q96G03 | PGM2 | Phosphoglucomutase-2 | 101041.3 |
| Q9H4Q4 | PRDM12 | PR domain zinc finger protein 12 | 132286.3 |
| Q9Y2W2 | WBP11 | WW domain-binding protein 11 | 188487.3 |
| Q7L8C5 | SYT13 | Synaptotagmin-13 | 41554 |
| Q9H0J9 | PARP12 | Protein mono-ADP-ribosyltransferase PARP12 | 230731.3 |
| P46783 | RPS10 | 40S ribosomal protein S10 | 94111.67 |
| O95461 | LARGE1 | LARGE xylosyl- and glucuronyltransferase 1 | 240975 |
| Q86T90 | KIAA1328 | Protein hinderin | 50061 |
| Q9HCM7 | FBRSL1 | Fibrosin-1-like protein | 54148.67 |
| P13682 | ZNF35 | Zinc finger protein 35 | 58519.67 |
| Q86VP6 | CAND1 | Cullin-associated NEDD8-dissociated protein 1 | 38794 |
| Q96EG3 | ZNF837 | Zinc finger protein 837 | 99687.67 |
| **Uniport accession number** | **Gene symbol** | **Protein name** | **Average signal intensity** |
| P98174 | FGD1 | FYVE, RhoGEF and PH domain-containing protein 1 | 54200.33 |
| Q96AQ6 | PBXIP1 | Pre-B-cell leukemia transcription factor-interacting protein 1 | 59759.67 |
| Q8IUX7 | AEBP1 | Adipocyte enhancer-binding protein 1 | 322847.3 |
| Q7Z7A1 | CNTRL | Centriolin | 203377.3 |
| P04114 | APOB | Apolipoprotein B-100 | 156562 |
| O75445 | USH2A | Usherin | 119697.7 |
| Q8N687 | DEFB125 | Beta-defensin 125 | 86537.67 |
| P32121 | ARRB2 | Beta-arrestin-2 | 463322.7 |
| Q92888 | ARHGEF1 | Rho guanine nucleotide exchange factor 1 | 149777.3 |
| Q5TCY1 | TTBK1 | Tau-tubulin kinase 1 | 106959.7 |
| Q86UK5 | EVC2 | Limbin | 375986.7 |
| Q9P2K1 | CC2D2A | Coiled-coil and C2 domain-containing protein 2A | 454176.7 |
| Q9Y483 | MTF2 | Metal-response element-binding transcription factor 2 | 259420 |
| P23470 | PTPRG | Receptor-type tyrosine-protein phosphatase gamma | 577316.7 |
| O95477 | ABCA1 | Phospholipid-transporting ATPase ABCA1 | 222803.3 |
| Q14517 | FAT1 | Protocadherin Fat 1 | 378816.7 |
