# Supplementary material for: Serum Angiopoietin-Like Protein 4: A Potential Prognostic Biomarker for Prediction of Vascular Invasion and Lymph Node Metastasis in Cholangiocarcinoma Patients
Source: Front Public Health. 2022 Mar 22;10:836985. doi: 10.3389/fpubh.2022.836985 (PMC8980351; doi:10.3389/fpubh.2022.836985)
Supplement: Supplementary file 4 [file Table_4.DOCX]

Supplementary Material

**Table S4.** Identified proteins in Group B patients of LC-MS/MS analysis

| **Uniport accession number** | **Gene symbol** | **Protein name** | **Average signal intensity** |
| --- | --- | --- | --- |
| B1AJZ9 | FHAD1 | Forkhead-associated domain-containing protein 1 | 220445 |
| O14974 | PPP1R12A | Protein phosphatase 1 regulatory subunit 12A | 128690.7 |
| O15021 | MAST4 | Microtubule-associated serine/threonine-protein kinase 4 | 1183807 |
| O15041 | SEMA3E | Semaphorin-3E | 144227 |
| O43147 | SGSM2 | Small G protein signaling modulator 2 | 108311.3 |
| O43155 | FLRT2 | Leucine-rich repeat transmembrane protein FLRT2 | 175362.3 |
| O43520 | ATP8B1 | Phospholipid-transporting ATPase IC | 41065 |
| O60281 | ZNF292 | Zinc finger protein 292 | 31375.33 |
| O60293 | ZFC3H1 | Zinc finger C3H1 domain-containing protein | 56107.73 |
| O60312 | ATP10A | Phospholipid-transporting ATPase VA | 18100.67 |
| O60841 | EIF5B | Eukaryotic translation initiation factor 5B | 22674.33 |
| O75054 | IGSF3 | Immunoglobulin superfamily member 3 | 122072.7 |
| O75077 | ADAM23 | Disintegrin and metalloproteinase domain-containing protein 23 | 29483 |
| O75306 | NDUFS2 | NADH dehydrogenase [ubiquinone] iron-sulfur protein 2 | 44446 |
| O94964 | SOGA1 | Protein SOGA1 | 305857.8 |
| P07942 | LAMB1 | Laminin subunit beta-1 | 90218.33 |
| P11473 | VDR | Vitamin D3 receptor | 44378.67 |
| P13569 | CFTR | Cystic fibrosis transmembrane conductance regulator | 57828.33 |
| P15918 | RAG1 | V(D)J recombination-activating protein 1 | 44112.67 |
| P16383 | GCFC2 | Intron Large complex component GCFC2 | 42777.47 |
| P18858 | LIG1 | DNA ligase 1 | 54736.27 |
| P35556 | FBN2 | Fibrillin-2 | 131926.3 |
| P35557 | GCK | Hexokinase-4 | 105125 |
| P46100 | ATRX | Transcriptional regulator ATRX | 96814.33 |
| P49368 | CCT3 | T-complex protein 1 subunit gamma | 345213.3 |
| **Uniport accession number** | **Gene symbol** | **Protein name** | **Average signal intensity** |
| P51815 | ZNF75D | Zinc finger protein 75D | 41098.67 |
| P54284 | CACNB3 | Voltage-dependent L-type calcium channel subunit beta-3 | 38101.93 |
| P78347 | GTF2I | General transcription factor II-I | 53656.67 |
| P78524 | DENND2B | DENN domain-containing protein 2B | 59973.23 |
| Q02952 | AKAP12 | A-kinase anchor protein 12 | 179379 |
| Q05519 | SRSF11 | Serine/arginine-rich splicing factor 11 | 33205.67 |
| Q13217 | DNAJC3 | DnaJ homolog subfamily C member 3 | 32298.33 |
| Q13591 | SEMA5A | Semaphorin-5A | 101072.7 |
| Q14563 | SEMA3A | Semaphorin-3A | 94870 |
| Q4J6C6 | PREPL | Prolyl endopeptidase-like | 147479.7 |
| Q4KMZ1 | IQCC | IQ domain-containing protein C | 147898.7 |
| Q5HYC2 | KIAA2026 | Uncharacterized protein KIAA2026 | 245455.3 |
| Q5M7Z0 | RNFT1 | E3 ubiquitin-protein ligase RNFT1 | 49806.53 |
| Q66GS9 | CEP135 | Centrosomal protein of 135 kDa | 151689.3 |
| Q6BDS2 | UHRF1BP1 | UHRF1-binding protein 1 | 141379.3 |
| Q6DJT9 | PLAG1 | Zinc finger protein PLAG1 | 140963.7 |
| Q6NUI6 | CHADL | Chondroadherin-like protein | 48308.33 |
| Q6P1N0 | CC2D1A | Coiled-coil and C2 domain-containing protein 1A | 106577.7 |
| Q6PL18 | ATAD2 | ATPase family AAA domain-containing protein 2 | 141975.3 |
| Q6TFL3 | CCDC171 | Coiled-coil domain-containing protein 171 | 68977.67 |
| Q6UVM3 | KCNT2 | Potassium channel subfamily T member 2 | 1043465 |
| Q6ZNG1 | ZNF600 | Zinc finger protein 600 | 69748 |
| Q6ZS11 | RINL | Ras and Rab interactor-like protein | 103423.3 |
| Q6ZUX3 | TOGARAM2 | TOG array regulator of axonemal microtubules protein 2 | 36876 |
| Q76M96 | CCDC80 | Coiled-coil domain-containing protein 80 | 106950 |
| Q7KYR7 | BTN2A1 | Butyrophilin subfamily 2 member A1 | 291614.3 |
| Q7Z570 | ZNF804A | Zinc finger protein 804A | 63217.33 |
| Q7Z572 | SPATA21 | Spermatogenesis-associated protein 21 | 110417.3 |
| Q86VI3 | IQGAP3 | Ras GTPase-activating-like protein IQGAP3 | 128136.8 |
| Q86VW0 | SESTD1 | SEC14 domain and spectrin repeat-containing protein 1 | 54928 |
| Q86X52 | CHSY1 | Chondroitin sulfate synthase 1 | 88776.67 |
| **Uniport accession number** | **Gene symbol** | **Protein name** | **Average signal intensity** |
| Q86XH1 | DRC11 | Dynein regulatory complex protein 11 | 164577.2 |
| Q86Z14 | KLB | Beta-klotho | 26290.6 |
| Q8IVT2 | MISP | Mitotic interactor and substrate of PLK1 | 69093 |
| Q8IXB1 | DNAJC10 | DnaJ homolog subfamily C member 10 | 26683.5 |
| Q8IYD9 | LAS2 | Lung adenoma susceptibility protein 2 | 187183 |
| Q8N1Y9 | N/A | Putative uncharacterized protein FLJ37218 | 63683 |
| Q8N6G6 | ADAMTSL1 | ADAMTS-like protein 1 | 269300 |
| Q8NB46 | ANKRD52 | Serine/threonine-protein phosphatase 6 regulatory ankyrin repeat subunit C | 131952 |
| Q8NB91 | FANCB | Fanconi anemia group B protein | 200433.3 |
| Q8NC56 | LEMD2 | LEM domain-containing protein 2 | 220825.3 |
| Q8NI51 | CTCFL | Transcriptional repressor CTCFL | 334031.3 |
| Q8TA94 | ZNF563 | Zinc finger protein 563 | 111936 |
| Q8TBP0 | TBC1D16 | TBC1 domain family member 16 | 83127.33 |
| Q8WTR8 | NTN5 | Netrin-5 | 64622 |
| Q8WWK9 | CKAP2 | Cytoskeleton-associated protein 2 | 55678.33 |
| Q8WWL7 | CCNB3 | G2/mitotic-specific cyclin-B3 | 125965.7 |
| Q92832 | NELL1 | Protein kinase C-binding protein NELL1 | 80979 |
| Q92974 | ARHGEF2 | Rho guanine nucleotide exchange factor 2 | 239833.3 |
| Q93009 | USP7 | Ubiquitin carboxyl-terminal hydrolase 7 | 601131.9 |
| Q93074 | MED12 | Mediator of RNA polymerase II transcription subunit 12 | 133432 |
| Q96AH8 | RAB7B | Ras-related protein Rab-7b | 66095 |
| Q96ES7 | SGF29 | SAGA-associated factor 29 | 90094 |
| Q96IC2 | REXO5 | RNA exonuclease 5 | 23072.2 |
| Q96M43 | NBPF4 | Neuroblastoma breakpoint family member 4 | 13095.3 |
| Q96PF1 | TGM7 | Protein-glutamine gamma-glutamyltransferase Z] | 72143.33 |
| Q99572 | P2RX7 | P2X purinoceptor 7 | 133262 |
| Q9BPX3 | NCAPG | Condensin complex subunit 3 | 147898.3 |
| Q9BRD0 | BUD13 | BUD13 homolog | 74068.67 |
| Q9BUQ8 | DDX23 | Probable ATP-dependent RNA helicase DDX23 | 47488.67 |
| Q9BX69 | CARD6 | Caspase recruitment domain-containing protein 6 | 165864.3 |
| **Uniport accession number** | **Gene symbol** | **Protein name** | **Average signal intensity** |
| Q9BXR5 | TLR10 | Toll-like receptor 10 | 83779.67 |
| Q9BY76 | ANGPTL4 | Angiopoietin-like protein 4 | 893416.7 |
| Q9BZC7 | ABCA2 | ATP-binding cassette sub-family A member 2 | 15797.33 |
| Q9C099 | LRRCC1 | Leucine-rich repeat and coiled-coil domain-containing protein 1 | 197373.3 |
| Q9C0D5 | TANC1 | Protein TANC1 | 193322.4 |
| Q9C0H5 | ARHGAP39 | Rho GTPase-activating protein 39 | 120425 |
| Q9C0J8 | WDR33 | pre-mRNA 3' end processing protein WDR33 | 565797 |
| Q9GZS0 | DNAI2 | Dynein axonemal intermediate chain 2 | 88169.33 |
| Q9H0A0 | NAT10 | RNA cytidine acetyltransferase | 35326 |
| Q9H2P0 | ADNP | Activity-dependent neuroprotector homeobox protein | 77843.67 |
| Q9HC96 | CAPN10 | Calpain-10 | 93694 |
| Q9NRY4 | ARHGAP35 | Rho GTPase-activating protein 35 | 50477.33 |
| Q9NUQ3 | TXLNG | Gamma-taxilin | 607424.7 |
| Q9NWS1 | PARPBP | PCNA-interacting partner | 15350 |
| Q9NXL9 | MCM9 | DNA helicase MCM9 | 130992.7 |
| Q9NZU7 | CABP1 | Calcium-binding protein 1 | 177652.7 |
| Q9P227 | ARHGAP23 | Rho GTPase-activating protein 23 | 47956.33 |
| Q9P291 | ARMCX1 | Armadillo repeat-containing X-linked protein 1 | 187819 |
| Q9UDY2 | ZO2 | Tight junction protein ZO-2 | 227718.3 |
| Q9UF56 | FBXL17 | F-box/LRR-repeat protein 17 | 26169.33 |
| Q9UKF2 | ADAM30 | Disintegrin and metalloproteinase domain-containing protein 30 | 132702 |
| Q9ULI0 | ATAD2B | ATPase family AAA domain-containing protein 2B | 109772.3 |
| Q9UM54 | MYO6 | Unconventional myosin-VI | 44553.07 |
| Q9Y2J4 | AMOTL2 | Angiomotin-like protein 2 | 132872 |
| Q9Y2K1 | ZBTB1 | Zinc finger and BTB domain-containing protein 1 | 159959 |
| Q9Y6K1 | DNMT3A | DNA (cytosine-5)-methyltransferase 3A | 97791.33 |
