# Supplementary figures and images for: Serum Angiopoietin-Like Protein 4: A Potential Prognostic Biomarker for Prediction of Vascular Invasion and Lymph Node Metastasis in Cholangiocarcinoma Patients
Source: Front Public Health. 2022 Mar 22;10:836985. doi: 10.3389/fpubh.2022.836985 (PMC8980351; doi:10.3389/fpubh.2022.836985)

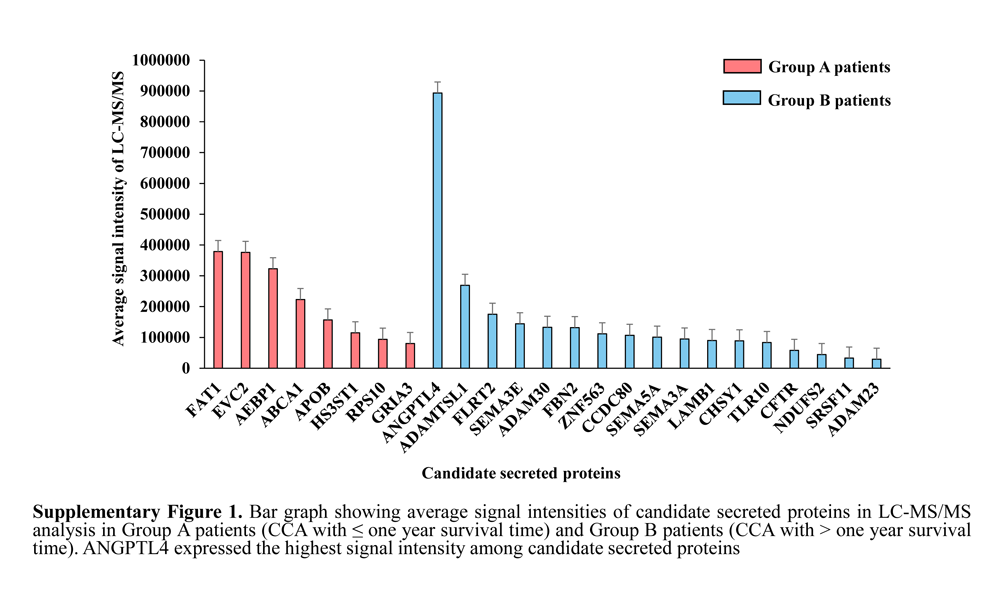

Supplement: Supplementary file 5 [file Image_1.TIF]

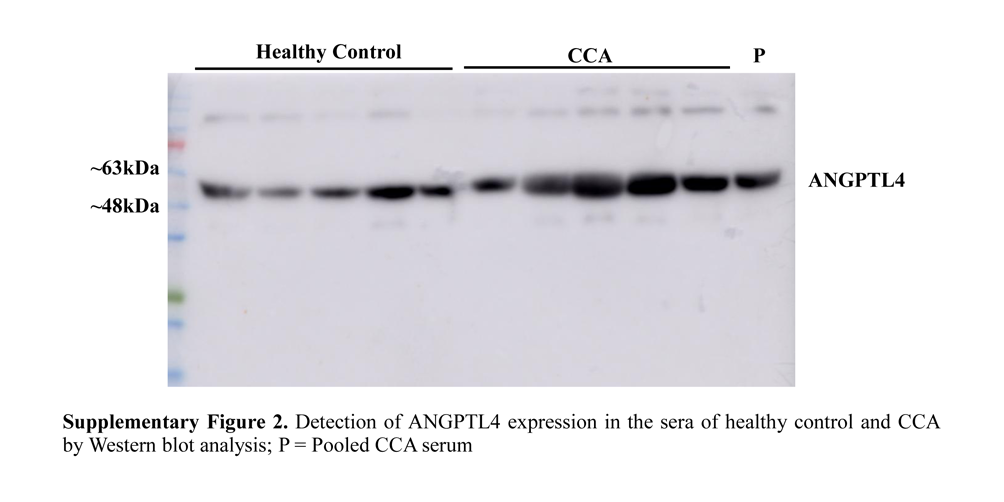

Supplement: Supplementary file 6 [file Image_2.TIF]

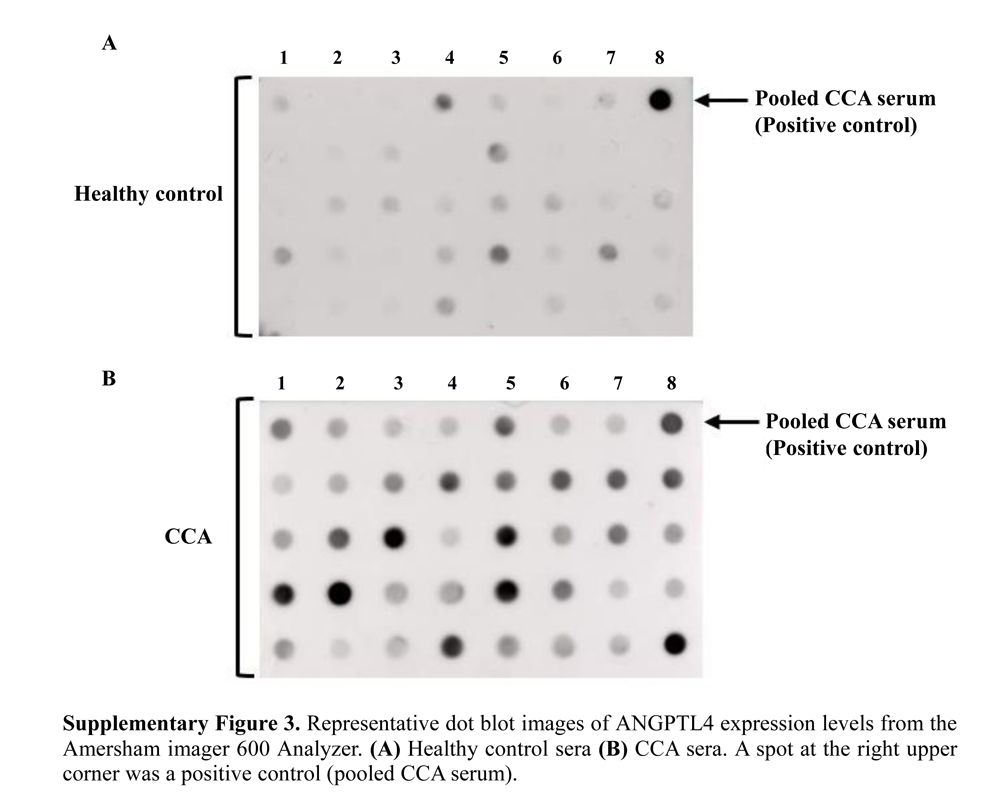

Supplement: Supplementary file 7 [file Image_3.TIF]

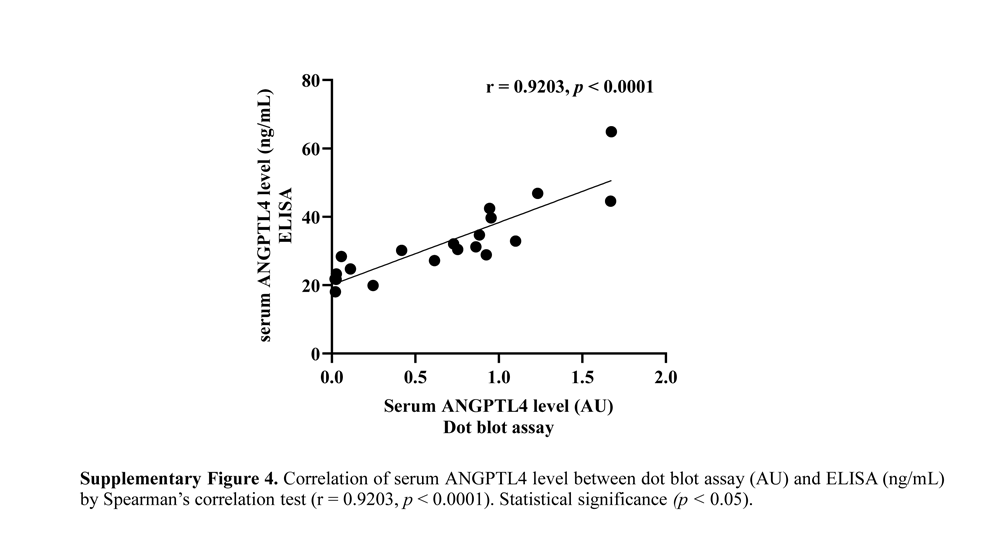

Supplement: Supplementary file 8 [file Image_4.TIF]

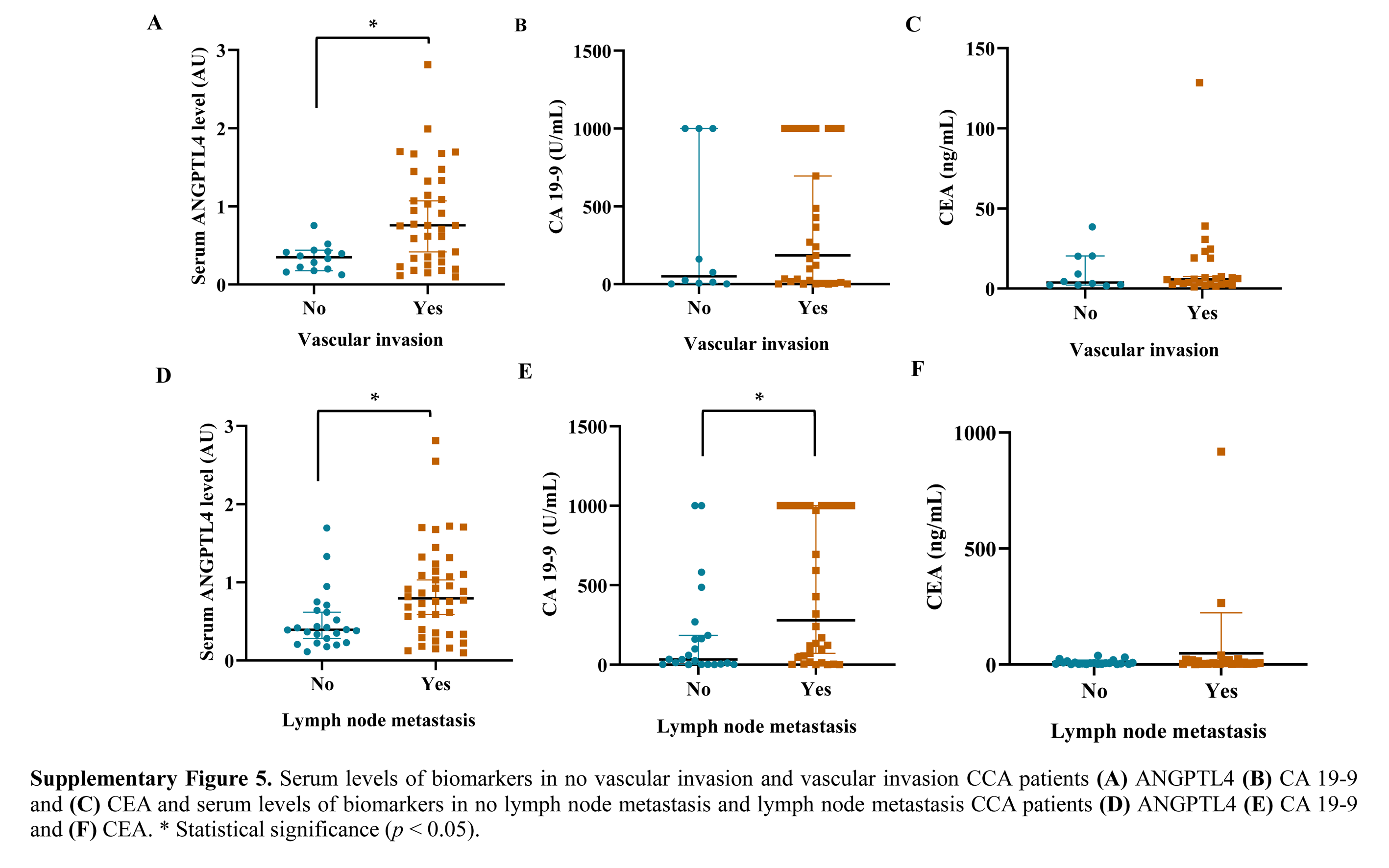

Supplement: Supplementary file 9 [file Image_5.TIF]
